# Uniformly Nanopatterned Graphene Field-Effect Transistors with Enhanced Properties

**DOI:** 10.1186/s11671-015-0976-2

**Published:** 2015-07-11

**Authors:** Duyoung Choi, Cihan Kuru, Youngjin Kim, Gunwoo Kim, Taekyoung Kim, Renkun Chen, Sungho Jin

**Affiliations:** Materials Science and Engineering, University of California, San Diego, La Jolla, CA 92093 USA; Department of Mechanical & Aerospace Engineering, University of California, San Diego, La Jolla, CA 92093 USA

**Keywords:** Graphene, Nanopatterned graphene, AAO, Nanopatterning, Field-effect transistor, Bandgap

## Abstract

We have successfully fabricated and characterized highly uniform nanopatterned graphene (NPG). Thin anodized aluminum oxide nanomask was prepared by facile self-assembly technique without using polymer buffer layer, which was utilized as a direct-contact template for oxygen plasma etch to produce near-periodic, small-neck-width NPG. The NPG exhibits a homogeneous mesh structure with an average neck width as small as ~11 nm. The highly uniform 11-nm neck width creates a quantum confinement in NPG, which has led to a record bandgap opening of ~200 meV in graphene for the given level of neck width. Electronic characterization of single-layer NPG field-effect transistors (FETs) was performed, which demonstrated a high on-off switching ratio. We found that the NPG allows for experimental confirmation of the relationship between electrical conductance and bandgap. This work also demonstrates that our direct-contact, self-assembled mask lithography is a pathway for low-cost, high-throughput, large-scale nanomanufacturing of graphene nanodevices.

## Background

Graphene has recently emerged as a new and exciting 2D material due to its remarkable properties including high charge mobility, mechanical strength, and flexibility [1–3]. Potential applications of graphene as electrodes in a wide range of devices including field-effect transistors (FETs) [4], touch-sensitive screens [5], liquid-crystal displays [6], light-emitting diodes [7], dye-sensitized solar cells [8, 9], and organic solar cells [10] have been reported.

However, due to the semimetallic nature of graphene, it lacks a bandgap, which is necessary for technological applications such as FETs. Hence, this results in a very low on/off ratio in graphene field-effect transistor devices. For practical applications, an on/off ratio on the order of 10^5^ is needed. One way to open a bandgap in graphene is to create geometrical constrictions of graphene material. This will lead to the confinement of electrons thus opening a bandgap. In order to increase the driving current for practical applications, such geometrical constrictions need to be maximized by fabrication of dense, ordered nanoribbon arrays, which has been achieved by electron-beam lithography [11, 12]. Although conventional lithographic methods can provide precisely located nanoarrays, the e-beam lithography is time-consuming and costly, and therefore, the size of the patterned area is often limited to the micrometer-scale regions.

To advance a facile process technique for nanopatterned graphene (NPG), we have specifically utilized an anodic aluminum oxide (AAO) lithography as it can be scaled to large-area substrates with high fidelity of patterning, which can be compatible with conventional lithographic processes [13, 14]. With an array of nanoholes introduced, the sheet resistance obviously becomes deteriorated due to a lost material pathway. Zeng et al. reported that nanometer-sized features on graphene cannot be achieved simply by directly placing the AAO membrane on reduced graphene oxide because of the rigid nature of AAO. Thus, polymethylmethacrylate (PMMA) was employed in their experiment as an adhesion layer between the AAO and graphene [14]. In that work, graphene nanomesh (GNM) with a neck width of 14.7 nm was produced, but the low density of nanoholes and the low number of on/off ratio failed to open up the bandgap of GNM for FET operation. The use of polymer buffer/adhesion layer causes less intimate contacts of the mask with the underlying graphene surface, so the resolution of the plasma etch nanopatterning can be adversely affected. However, in the present research, we were able to overcome the issue by fabricating a less rigid and thin AAO template (~200-nm thick), and successfully demonstrated fabrication of nanohole-patterned graphene using this oxide template without polymer buffer layer thus avoiding such complicated processes. Moreover, our NPG is semiconducting in behavior with a substantially increased effective energy gap of 200 MeV at room temperature. The key to this success was the thinness and uniformity of the AAO membrane that we provided in fabrication of water-floatable AAO membranes.

Here, we report the production of a graphene nanostructure that can open up a bandgap in a large sheet of single-layer graphene (SLG). We focus on experimental investigations in SLG FETs and the implications for the device performances. The patterned graphene is prepared by self-assembled mask lithography using a floating AAO that can be placed on the device surface by lift-up of the device from underneath. Such nanostructuring process can effectively open up a conduction bandgap in a large piece of graphene, e.g., by using a several-centimeter-sized AAO membrane. We expect that the relative ease of our AAO lithography technique which can be implemented and scaled to large areas, together with the demonstrated effectiveness in controlling the electronic properties of graphene, will be useful for efforts toward practical large area, commercial applications of graphene in electronics, thin-film devices, flexible electronics, optoelectronics, and sensing.

## Methods

### Preparation of AAO Membrane

A 0.5-mm thick annealed Al foil purchased from Alfar Aesar (99.99 %) was used as the starting material. The Al foil was successively degreased by acetone and isopropyl alcohol with ultrasonication, followed by deionized (DI) water rinse and nitrogen gas blow. The Al foil was slightly etched in a 1 M NaOH aqueous solution to remove any possible surface contaminations prior to surface-smoothing electropolishing process conducted at 20 V in a solution of perchloric acid (70 %) and ethanol (99.9 %) (1:4 volume ratio) at 5 °C for 15 min, using a Pt counter electrode. Then, a two-step anodization process of the Al foil was carried out by incorporating the Al foil as the working electrode and Pt as the counter electrode, immersed in 0.3-m oxalic acid. The electrolyte temperature was maintained at 1 °C during the anodization process using a powerful refrigeration bath (RTE7, Thermo Scientific) in which the coolant circulates a double-wall glass chamber. After the first anodizing process, which took about 3 h at an operating voltage of 40 V, the anodized Al foil was immersed for 1 h in a mixed solution of phosphoric acid (6 wt%) and chromic acid (1.8 wt%) kept at 75 °C to remove the alumina layer formed in the first anodizing step. The second anodizing step was implemented for 10 min while other experimental conditions were unchanged compared with the first anodizing step, in order to form an ordered porous alumina membrane on the Al foil. Then, the Al metallic substrate underneath the AAO layer was selectively removed with a mixed HCl and CuCl_2_ solution for 10 min. Any residual Cu debris adhered to the bottom of the AAO barrier layer was removed by placing the sample in nitric acid for a few seconds and washed in DI water immediately after. The barrier layer in the bottom of the AAO holes was then removed by a 5 wt% phosphoric acid etching for 10 min to 2 h.

### Fabrication of NPG

A single-layer graphene was purchased from ACS material (MA, USA). Before graphene on Cu backing layer was separated and transferred to other substrates, the back side of graphene was first removed by oxygen plasma. The top side of graphene was protected by a PMMA layer coating during the O_2_ plasma etching. The graphene film was then transferred onto a 300-nm SiO_2_-coated Si substrate (Si/SiO_2_) using chemical processing steps. The chemical process for graphene transfer consists of the etching of Cu foil, transferring the floating graphene onto a Si/SiO_2_ substrate by lift-up in an aqueous solution bath, followed by washing with water, acetone, and isopropyl alcohol as described elsewhere [9]. After that, the PMMA layer was removed by dissolving it in acetone. Furthermore, the rapid thermal annealing was carried out for graphene placed on the Si/SiO_2_ substrate by heating to 400 °C under a N_2_ atmosphere to remove the residual PMMA and promote the adhesion between graphene and the oxide layer.

The prepared AAO template floating in water was placed on the graphene as an etch mask by lifting up the Si/SiO_2_ substrate from underneath. After that, the sample was annealed in a vacuum at 180 °C for 2 h, in order to allow the AAO membrane to stick tightly on the graphene surface. Then, oxygen plasma (30 W, 150 mTorr) was applied through the AAO template holes to etch and create pores on the graphene. The details of recipes and procedures for the formation of NPG were explained in previous study [13].

### Graphene Characterization

The sample microstructure was characterized by ultra-high resolution scanning electron microscopy (UHR SEM; FEI XL30). Raman spectroscopy was used as a nondestructive tool for probing the edges and the crystalline sp^2^-bonded structure of the graphene [15]. Raman spectra were collected using a Renishaw Raman spectrometer inbuilt with an Ar^+^ laser of a wavelength of 514 nm for quantifying the degree of structural order and charge transfer characteristics. To measure the sheet resistance of graphene nanomesh (GNM), Jandel Four-Point Probe was employed for the four-point measurement. The optical property of the graphene samples was characterized by UV/Vis spectrophotometer (UNICO SQ-4802).

## Results and Discussion

**Fig. 1:**
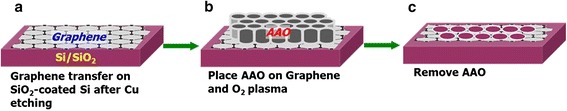
Schematic of nanopatterned graphene fabrication. **a** CVD-grown graphene was transferred onto a Si/SiO_2_ substrate. **b** An AAO template was placed on top of graphene. **c** Graphene in the exposed area was etched away by O_2_ plasma through the AAO pores, and then the AAO was removed. Finally, porous graphene on SiO_2_ was obtained

**Fig. 2:**
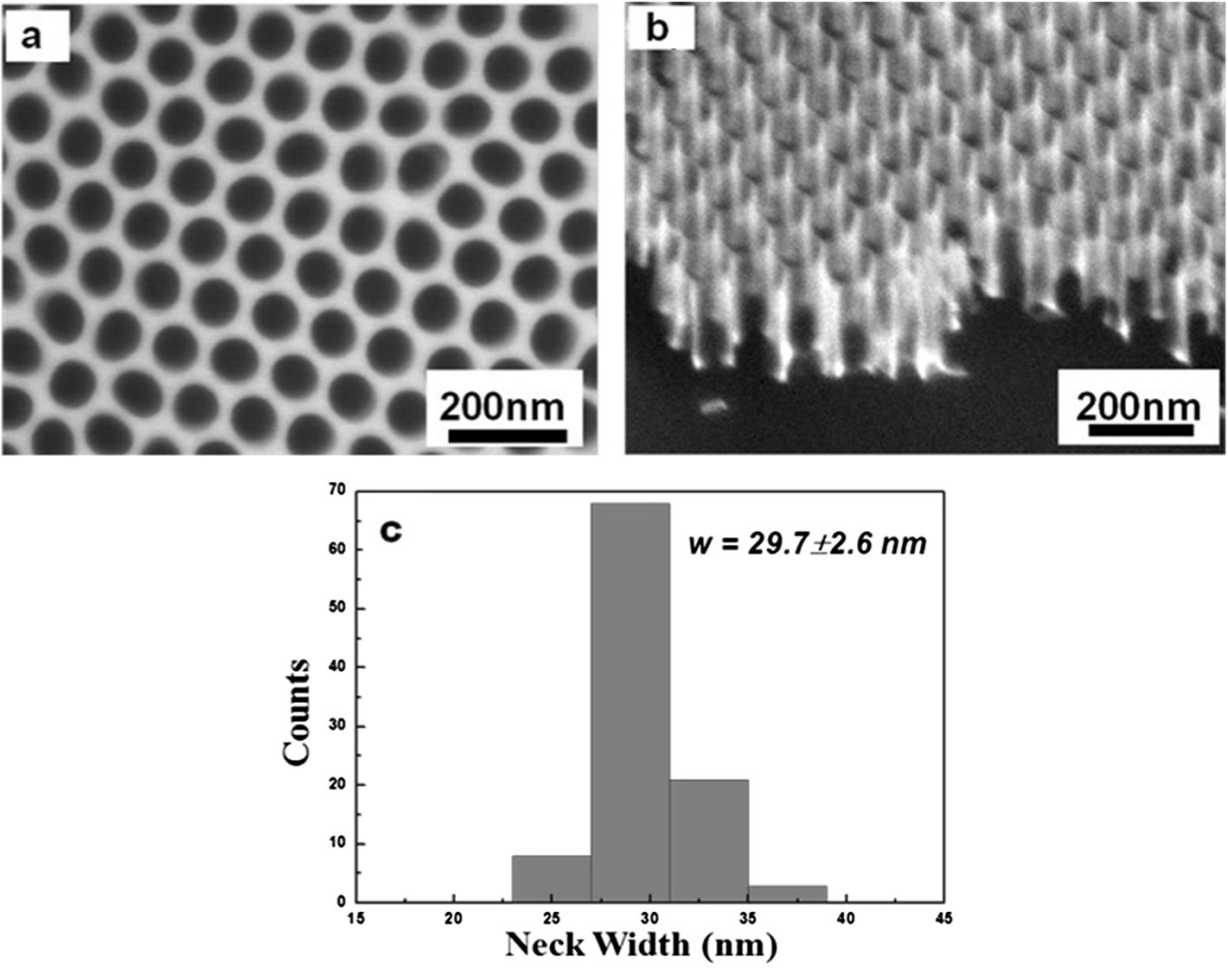
Scanning electron microscopy (SEM) images. **a** An AAO template (top view). **b** A tilted AAO membrane with a ~200-nm thickness. **c** Histogram of the neck width (*w*) between AAO pores with an average neck width of 29.7 nm (std. dev. ±2.6 nm)

Figure 1 schematically illustrates the present approach for fabricating NPG. The CVD-grown graphene on Cu foil was used as the starting material. The copper layer was removed by chemical reaction with an aqueous 0.1 M ammonium persulfate solution, (NH_4_)_2_S_2_O_8_. The floating graphene in water was transferred onto a Si/SiO_2_ substrate. We used the SiO_2_-coated Si (Si/SiO_2_) substrate for electrical measurements of the FET device. The AAO membrane was placed on graphene, and the transferred AAO membrane was used as the etch mask for the fabrication of NPG. After the oxide template was placed on top of graphene, O_2_ plasma etching was employed to generate nanopores in the graphene layer. Finally, the AAO mask was etched away by a NaOH solution, and the sample was washed with acetone. The AAO membrane prepared by a two-step anodization of high-purity aluminum foil and this self-assembly fabricated AAO membrane was used as a mask during the oxygen plasma etching of graphene for nanopore array formation (Fig. 2).

**Fig. 3:**
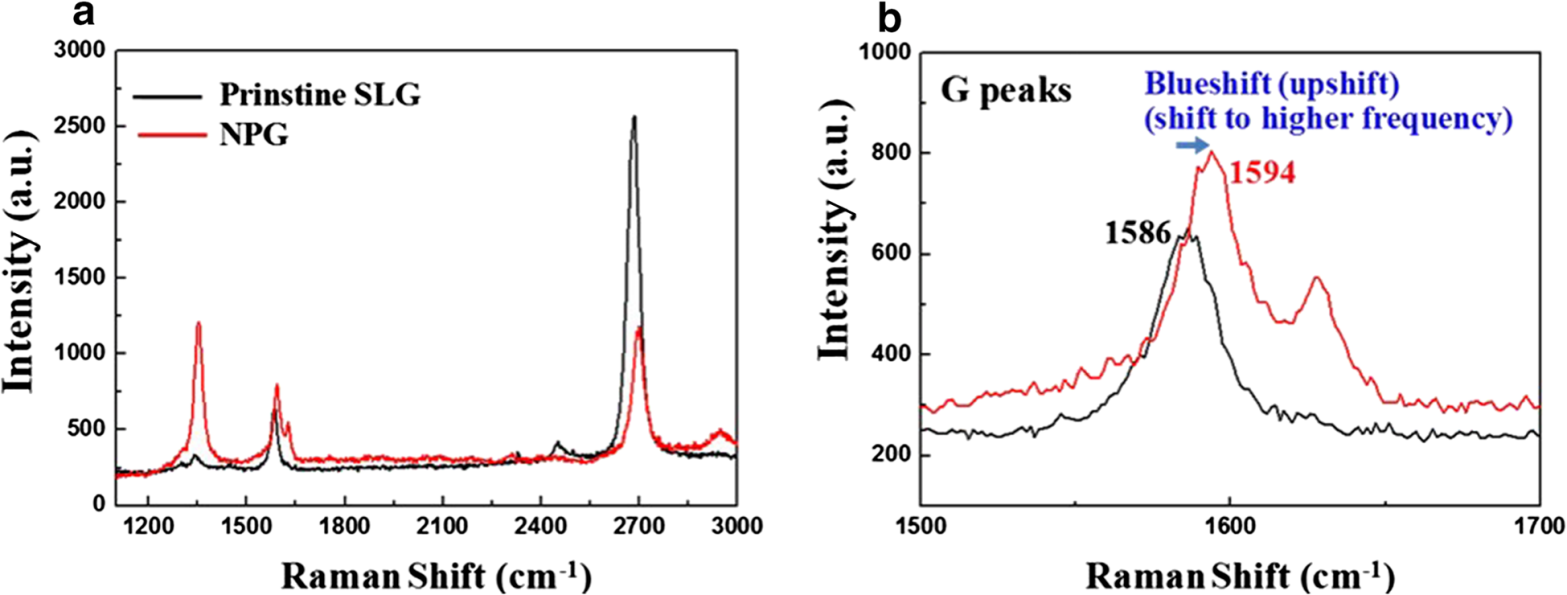
Comparison of Raman spectra. **a** Before versus **b** after patterning NPG showing ~8 cm^−1^ blueshift on G band due to nanopatterning (11–13-nm neck width)

Raman spectroscopy was used as a nondestructive tool for probing the edge structure and the crystallinity of sp^2^-bonded graphene. Figure 3 demonstrates the Raman spectra of pristine graphene and NPG. The Raman data was taken from different spots on graphene to check the uniformity. Prior to patterning, the G (~1586 cm^−1^) and 2D (~2682 cm^−1^) bands were prominent. The D peak at ~1341 cm^−1^ is related to defects and disorder. This is forbidden in perfect graphitic systems and requires a defect for its activation, and so is observed at the edges of graphene samples [15–17]. The integrated intensity ratio of the D band and G band (*I*_D_/*I*_G_) is a parameter sensitive to defect density [17, 18]. In Fig. 3a, the high D peak was observed on porous graphene with the value of *I*_D_/*I*_G_ increased by a factor of 3, which suggests that defects in our sample are significantly formed by nanopatterning and pore edge formation. After nanopatterning, there is a systematic upshift in the position of the G band. The G band position for porous graphene was observed at ~1594 cm^−1^, which can be compared with the G position of pristine graphene (~1586 cm^−1^) in our sample. This upshift in the G band position further confirms the hole doping in the NPG by the formation of oxygen dangling bonds with graphene, as reported by previous research [15]. We also note that there is an increase in the intensity ratio of the *I*_G_/*I*_2D_ with more defects. The increase in the *I*_G_/*I*_2D_ in NPG is due to the alteration of its electronic transformation from semimetallic to semiconducting with successive opening of bandgap [19].

**Fig. 4:**
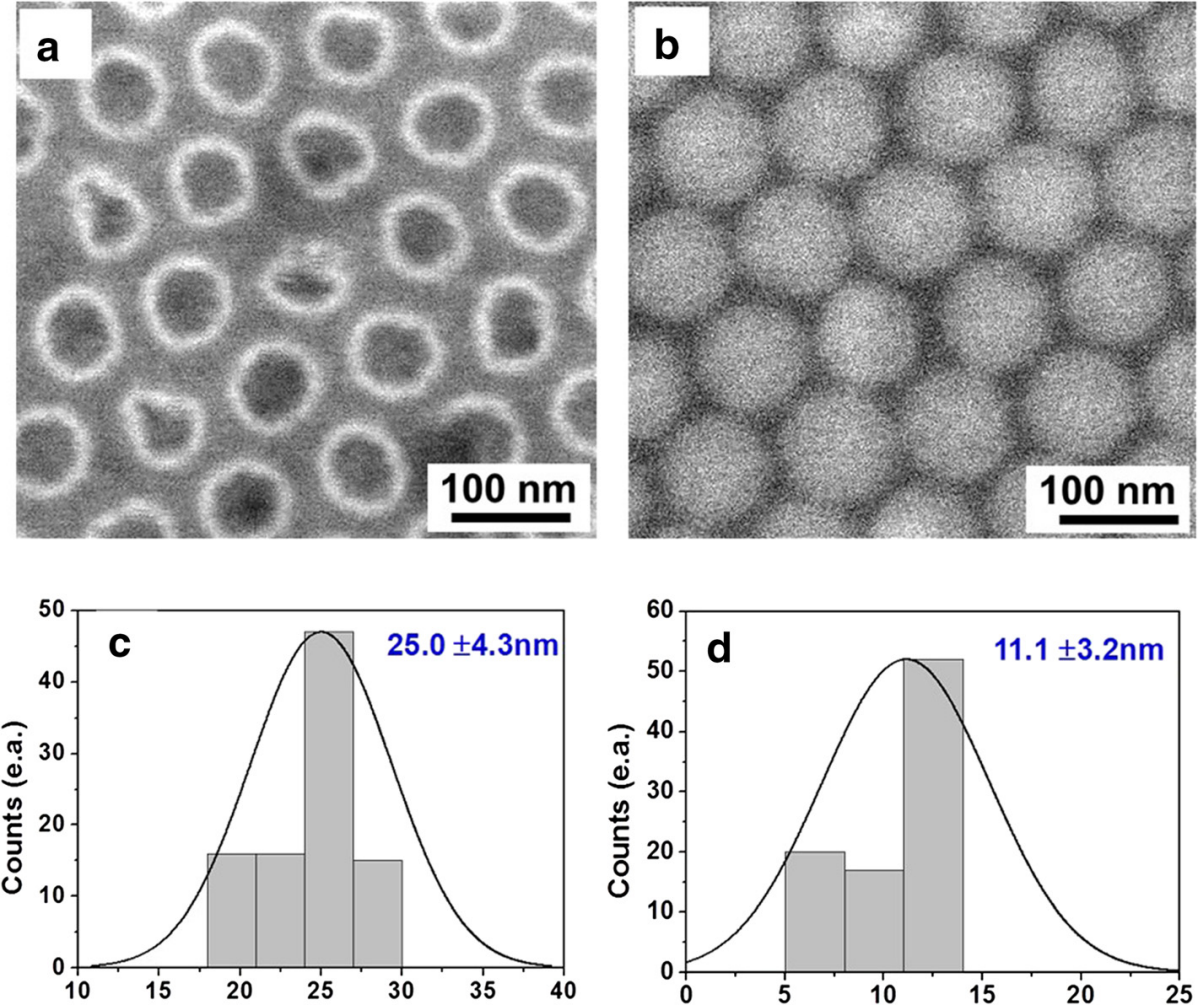
Example SEM images of the NPG surface after removing the AAO mask. **a** NPG with a 30-s etching time. **b** NPG with a 40-s etching time. **c** The neck width in **a** is 25.0 ± 4.3 nm. **d** The neck width in **b** is 11.1 ± 3.2 nm

Figure 4 shows some example SEM images of NPGs with different average neck widths with different etching times from 30 to 40 s. Furthermore, it is possible to tune the coverage areas of NPG by controlling the etching time. As the neck width represents the smallest dimension that controls charge transport through the system, we have carried out statistical analysis of the neck widths of the NPG obtained after the O_2_ etching (Fig. 4c, d). The histograms resulting from the statistical analysis show that the average neck width on graphene after controlled etching for 30 s is 25.0 ± 4.3 nm (Fig. 4c). It is expected that a neck-width reducing process, such as utilizing a controlled oxygen plasma etch, could be utilized, which can lead to a substantially reduced neck width and associated interesting change in the degree of a bandgap opening, creating a further enhanced quantum confinement effect. Figure 4d shows a NPG with a smaller average *w* of 11.1 ± 3.2 nm, which is achieved through an intentional slight over-etching by exposing to 40 s oxygen plasma. These SEM analyses on our graphene layer agreed with previous studies which clearly demonstrate that highly uniform porous graphene can be obtained with a controllable etching time by the template approach.

**Fig. 5:**
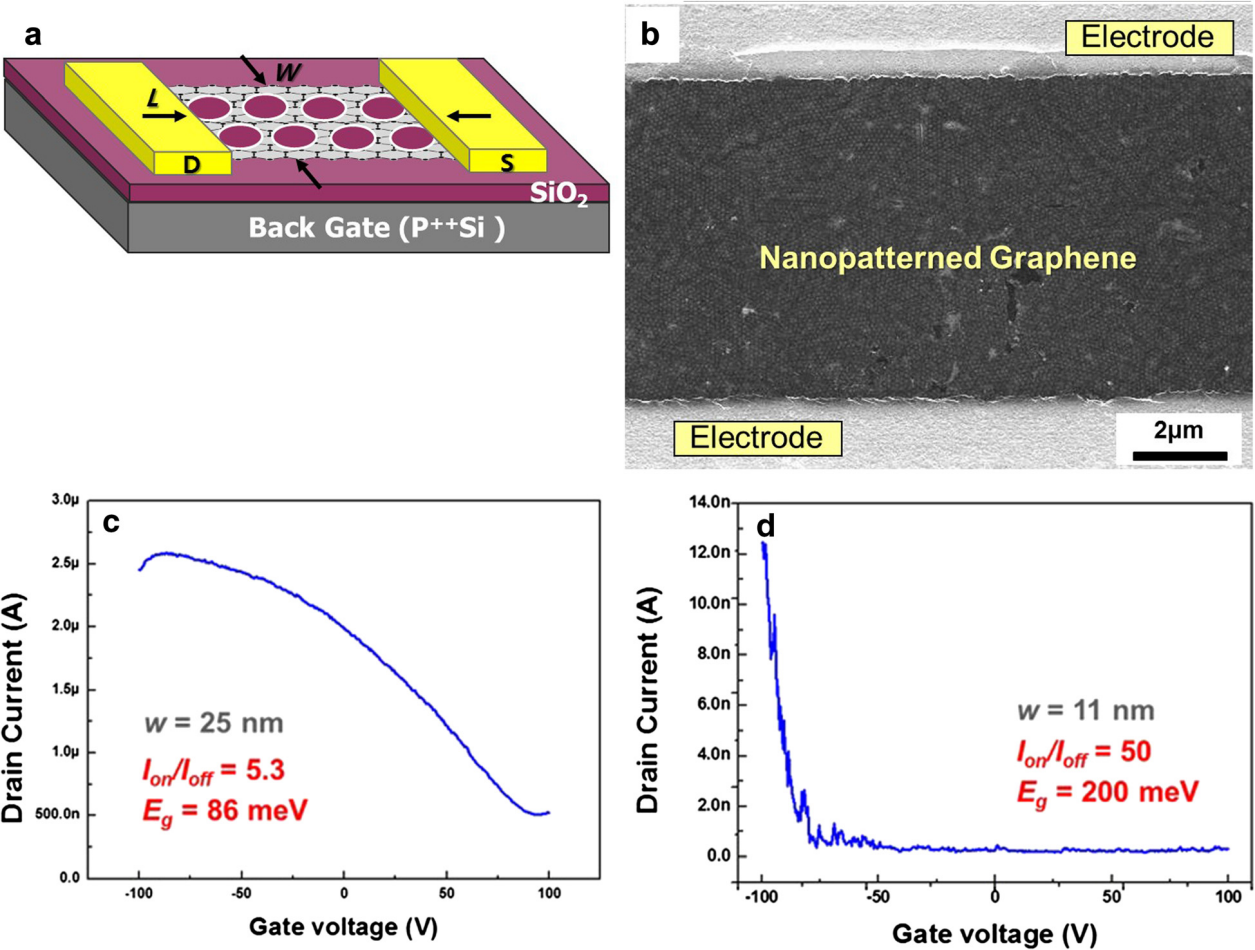
FET structure and electrical properties. **a** Schematic illustration of the FET device fabricated using the NPG. **b** SEM image showing the top view of the NPG-based FET device. **c** Drain current (*I*_d_) versus gate voltage (*V*_g_) for a FET device with *w* = 25.0 ± 4.3 nm. (The electronic measurement was carried out in ambient conditions at room temperature.) **d** *I*_d_ versus *V*_g_ for a device with *w* = 11.1 ± 3.2 nm

Figure 5 displays the electrical characteristics of field-effect transistors (FETs) containing the NPG structure at room temperature. Figure 5a schematically illustrates the structure of a patterned graphene FET device, in which a rectangular-shaped NPG with total channel width *W* and channel length *L* serves as the conduction channel. A pair of metallic pads (Ti/Au) serves as drain and source contacts. The 300-nm-thick thermal oxide SiO_2_ layer and degenerated (p^++^) Si wafer are used as the gate dielectric and the back gate, respectively. Figure 5c, d shows the electrical transport characteristics of a typical patterned graphene transistor with an average neck width of ~25 nm. Drain current (*I*_d_) versus gate voltage (*V*_g_) characteristics for the transistor show a typical p-channel transistor behavior (Fig. 5c, d). The increase in p-doping is likely due to increase in oxygen plasma exposure, resulting in dangling bonds on the edges of the holes [20]. The hole doping observed in the NPG is similar to that of graphene nanoribbon devices and can be attributed to edge oxidation in the O_2_ plasma process or physisorbed oxygen from the ambient and other species during the nanofabrication process.

The ability to control the NPG periodicity and neck width is very important for controlling their electronic properties because charge transport properties are highly dependent on the width of the critical current pathway. In the case of graphene nanoribbons, both theoretical and experimental works have shown that the size of the electronic bandgap is inversely proportional to the ribbon width [21, 22]. Therefore, we expect that the electronic bandgap of NPG inversely scales with the average ribbon width (i.e., *E*_g_ ~ *α*/*w*, and *α* is a coefficient with 0.95 nm eV for nanomesh) [23, 24]. Furthermore, the on/off current ratio of a FET device exponentially scales with the bandgap (i.e., (*I*_on_/*I*_off_ · exp(*E*_g_/*kT*), where *k* is Boltzmann constant and *T* is the absolute temperature) [24]. So the *I*_on_/*I*_off_ value of a NPG transistor is expected to inversely scale with the average neck width, as expressed in Eq. (1), where *C* is a dimensionless constant. Equation (1) can be simplified to Eq. (2) related to bandgap energy.

1 $$ {I}_{\mathrm{on}}/{I}_{\mathrm{off}}\approx {\mathrm{e}}^{\mathit{\mathsf{\alpha}}/\mathit{\mathsf{k}}\mathit{\mathsf{T}}\left(1/\mathit{\mathsf{w}}\right)}=\mathit{\mathsf{C}}{\mathrm{e}}^{\mathit{\mathsf{\alpha}}/\mathit{\mathsf{k}}\mathit{\mathsf{T}}\left(1/\mathit{\mathsf{w}}\right)} $$

2 $$ {E}_{\mathrm{g}}=kT\left[ \ln \left({I}_{\mathrm{on}}/{I}_{\mathrm{off}}\right)- \ln \left(\mathit{\mathsf{C}}\right)\right] $$

We have achieved the current on/off ratio values significantly higher than those in the previously reported FET devices of graphene nanoribbon (GNR) and graphene nanomesh (GNM) [21–25]. The expected bandgap from the relation of *E*_g_ ~ *α*/*w* by an average neck width of ~10 nm was 95 MeV. In Fig. 5d, however, the actual bandgap in our FET device with an 11-nm neck-width NPG is estimated to be ~200 MeV from Eq. (2) with our measured *I*_on_/*I*_off_ value of 50. By contrast, the FET device with a larger 25-nm neck-width NPG exhibits an order of magnitude smaller *I*_on_/*I*_off_ ratio of ~5.3 with much less bandgap opening as shown in Fig. 5. There is a difference between the calculated bandgap values from the relations with the neck width of our FET having an average neck width of ~11 nm and the on/off current ratio experimentally measured, with the actual measured ratio being higher. Further detailed study is in progress to understand the mechanism behind this observation of surprisingly highly effective bandgap in our NPG samples. We assume that the unusually high on/off ratio in our more extensively patterned graphene affected the bandgap opening, possibly due to the highly dense and uniform NPG nanostructure throughout the large-area samples. Such results point to a possibility of utilizing the properly and highly uniformly nanopatterned large-area graphene as promising electronic devices [26, 27].

Electrical characterization of NPG confirmed that the current on-off ratio is inversely proportional with the neck width, indicating the formation of an effective gap due to the confinement effect. We have shown that both electronic transport and Raman characteristics change in a concerted manner on graphene patterning. The availability of such well-controlled NPG structure will provide an interesting possibility for a more in-depth fundamental investigation of transport behavior in the highly interconnected graphene network, and will enable exciting opportunities in sensitive electronics and sensor devices.

## Conclusions

We demonstrate a successful fabrication of very fine dimension NPG using a thin-floating anodic aluminum oxide (AAO) membrane etching mask. The membrane was directly transferred onto a hydrophobic graphene surface for well-adhered and stacked manner on the graphene due to the van der Waals force, thus allowing a high-density, small-neck-width NPG structure fabrication, without using any intermediate buffer/adhesion polymer which could adversely affect the resolution of plasma etching patterning of graphene. The NPG so produced exhibits homogeneous mesh structures with an average neck width as small as ~11 nm. Electronic characterization of a single-layer NPG FET structure with an 11-nm neck width creates a quantum confinement in NPG, which has led to an impressive bandgap opening of ~200 MeV. The NPG structures with different neck widths allowed experimental confirmation of the relationship between electrical conductance and bandgap. Electrical characterization of the NPG-based FET device confirmed that the current on/off ratio is inversely proportional with the neck width, indicating the formation of an effective gap due to a confinement effect. The availability of such NPG will provide an interesting system for a more in-depth fundamental investigation of transport behavior in the highly interconnected, small-width graphene network and will enable exciting opportunities in sensitive electronic or sensor devices. This work also demonstrates that self-assembled mask lithography is a pathway for low-cost, high-throughput, large-scale nanomanufacturing of NPG with critical dimensions down to nanometer regime.
